# Epidemiological characteristics and spatial clustering analysis of human brucellosis in Zibo City, Shandong Province, China, 2006–2024

**DOI:** 10.3389/fpubh.2025.1580265

**Published:** 2025-06-27

**Authors:** Rongtao Zhao, Ruixuan Sun, Feng Zhang

**Affiliations:** Zibo Center for Disease Control and Prevention, Zibo, Shandong, China

**Keywords:** epidemiological characteristics, spatial clustering analysis, human brucellosis, seasonal, global spatial autocorrelation, temporal-spatial aggregation

## Abstract

**Background:**

The significant rise in human brucellosis incidence is a serious public health issue in Zibo City. However, its temporal and spatial distribution has not been thoroughly investigated.

**Objective:**

This study aims to describe the demographic, temporal, and spatial distribution patterns and clustering characteristics of human brucellosis cases in Zibo City from 2006 to 2024, in order to develop and implement effective scientific prevention and control strategies.

**Methods:**

Case data were obtained from the Infectious Disease Surveillance System of the Chinese Disease Control and Prevention Information System. The epidemiological characteristics and spatial aggregation of human brucellosis were analyzed using descriptive analysis and spatial autocorrelation analysis. The incidence of brucellosis in 2025 was predicted using an ARIMA model.

**Results:**

Data on human brucellosis cases in Zibo City from 2006 to 2024 were obtained from the national infectious disease reporting information management system. Spatial autocorrelation analysis and temporal-spatial scan statistics were used to identify potential changes in the temporal-spatial distribution of human brucellosis in Zibo City. From 2006 to 2021, a total of 2,176 brucellosis cases were reported in Zibo City, with an average annual incidence rate of 2.50 per 100,000. Middle-aged and older adult populations (aged 35–74 years) were the primary affected groups, accounting for 86.76% (1,888/2,176) of all reported cases. The incidence of brucellosis in Zibo City showed a long-term upward trend and exhibited significant seasonal variations, with peaks occurring between March and September each year. From 2006 to 2024, the incidence gradually expanded from the northern and central regions to the southern regions. Global spatial autocorrelation analysis revealed a positive correlation in brucellosis incidence between 2009 and 2012–2024. Spatiotemporal cluster analysis identified a primary cluster in the high-incidence areas of northern Zibo City, with four secondary clusters appearing in areas where brucellosis outbreaks had previously occurred. According to ARIMA model predictions, the monthly incidence rate of brucellosis in Zibo City has declined steadily from 0.75/100,000 in 2010 to near-zero levels by 2020 and is projected to remain extremely low through 2025.

**Conclusion:**

Human brucellosis remains a serious public health concern in Zibo City. Special monitoring and control plans and measures may be required for the high-incidence areas in northern Zibo. Additionally, epidemic response capabilities should be enhanced in high-incidence areas to prevent further increases in brucellosis incidence.

## Introduction

Brucellosis is a zoonotic disease caused by bacteria of the genus Brucella, with at least 500,000 human cases reported globally each year (1). This disease not only causes severe physical and mental harm, including disability, but also proves difficult to treat in chronic cases, increasing societal healthcare costs and depleting medical resources. Low- and middle-income countries are considered high-prevalence regions (2, 3). Zoonotic brucellosis is typically caused by various species of Brucella, with 12 known species identified so far (*Brucella melitensis, Brucella abortus, Brucella suis, Brucella canis, Brucella ceti, Brucella pinnipedialis, Brucella microti, Brucella inopinata, Brucella raniformis, Brucella pseudoinopinata, Brucella vulpis and Brucella papionis*). Among these, *B. melitensis* is the most prominent zoonotic pathogen. The occurrence of human brucellosis is directly related to the prevalence of the disease in animals within specific geographic regions. The primary sources of human Brucella infection are livestock such as cattle, sheep, and pigs. Humans contract brucellosis mainly through direct or indirect contact with infected animals or their products, as well as through the consumption of contaminated animal-derived foods (4, 5). Routes of transmission include the skin, digestive tract, and respiratory system. Brucellosis outbreaks not only pose a significant threat to human health but also hinder livestock production, thereby impacting the global economy and trade. Additionally, they raise concerns about food safety, making brucellosis a critical public health issue worldwide, particularly in developing countries (6, 7).

In recent years, the number of brucellosis cases in China has risen significantly, increasing from approximately 20,000 cases in 2006 to over 60,000 in 2020 (16). Although the epidemic remained active in high-risk regions from 2021 to 2023, with annual reported cases stabilizing between 50,000 and 60,000, sporadic clusters were observed in localized areas. Brucellosis has now been reported across all 31 provinces (autonomous regions) in China, emerging as a critical public health challenge (8–12). As a pivotal analytical methodology, spatial autocorrelation techniques have been extensively applied across socio-economic research domains (13). Spatial autocorrelation denotes the statistical interdependence of georeferenced variables within geographic proximity. Contemporary epidemiological studies have increasingly employed spatial analytical techniques in cross-disciplinary investigations of disease transmission patterns, notably exemplified by dengue fever (14) and pulmonary tuberculosis (15). There were also some studies on the use of spatial autocorrelation analysis for human brucellosis (16). Zhao (17) used spatiotemporal scanning statistics to explore the spatial–temporal aggregation of human brucellosis in in mainland China from 2012 to 2018. Yu (8) analyzed the spatial and temporal clustering of human brucellosis in Shandong Province, 2015–2021. Compared with the traditional method, spatial autocorrelation analysis is used to analyze the spatial epidemic characteristics of disease distribution, and the correlation of incidence rate in adjacent regions can be obtained. Spatial autocorrelation analysis can provide suggestions for the government to formulate and implement appropriate regional public health intervention strategies to prevent and control human brucellosis.

Recent epidemiological patterns indicate a highly endemic status with distinct spatiotemporal clustering, historically concentrated in northwestern and northeastern China but increasingly spreading to central and southern regions (18–21). Previous studies on brucellosis in Zibo City have focused on descriptive epidemiology, leaving its spatiotemporal distribution underexplored. This study systematically analyzes the epidemiological characteristics and spatiotemporal clustering of human brucellosis in Zibo City from 2006 to 2024, aiming to provide evidence for optimizing prevention and control strategies.

## Methods

### Laboratory diagnostic criteria

Case definitions followed the Diagnostic Criteria for Brucellosis (WS269-2019): Exposure history or clinical symptoms: Laboratory testing was required for individuals with suspected exposure or symptoms; Laboratory confirmation: A positive result in both the Rose Bengal Plate Agglutination Test (RBT) and the Standard Tube Agglutination Test (SAT: serum agglutination titer ≥1:100++, or ≥1:50++ for symptomatic patients with disease duration >1 year), or isolation of Brucella from blood, bone marrow, body fluids, or excreta (22, 23).

### Data sources

**Figure 1:**
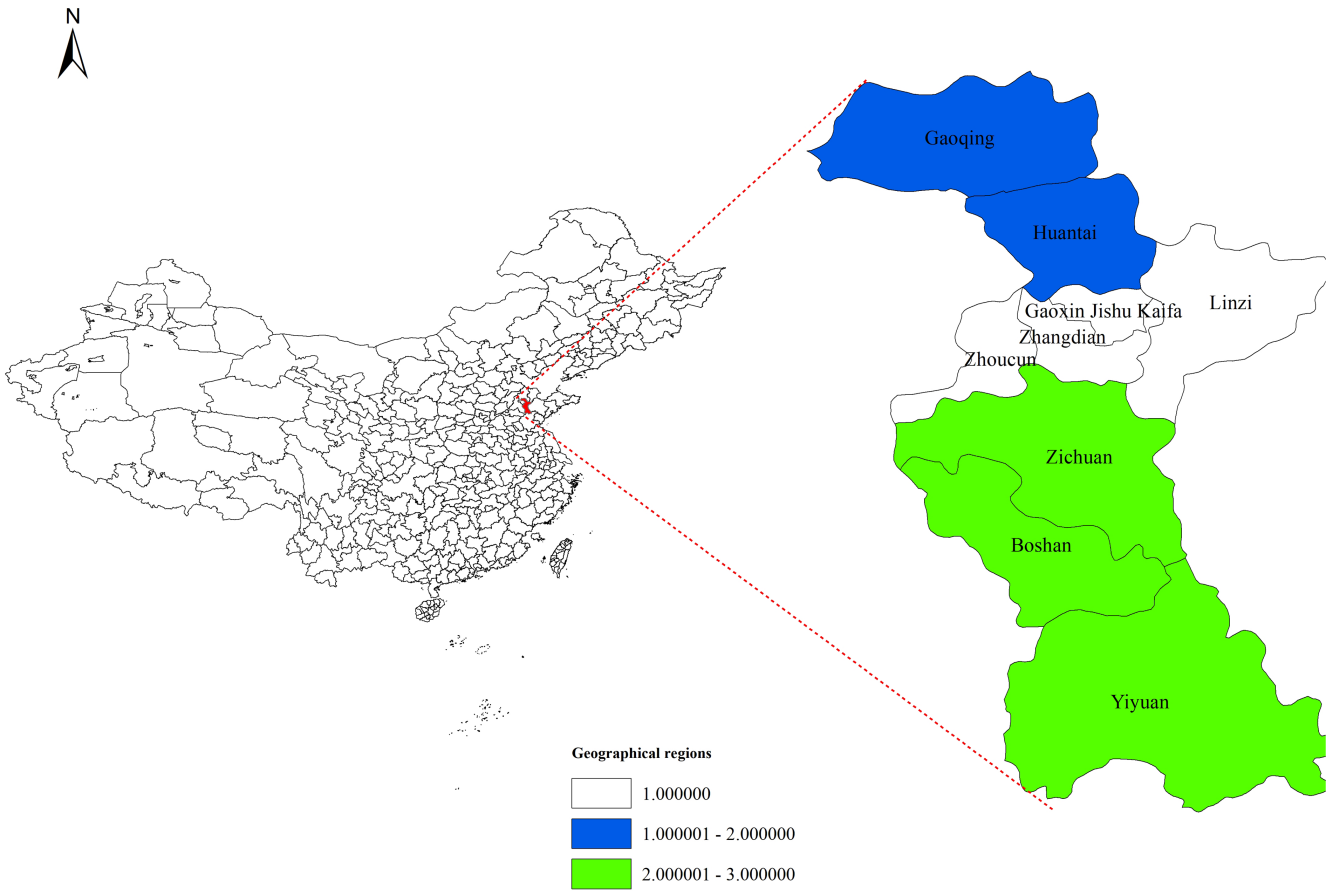
Tree geographical regions of Zibo City.

Human brucellosis is a Category B notifiable infectious disease in China. Confirmed cases must be reported to the National Infectious Disease Reporting Information Management System (NIDRIMS), established in 2004 and covering all healthcare institutions nationwide (24). This study extracted brucellosis case data for Zibo City (2006–2024) from NIDRIMS. Digital vector maps of Zibo City (Shandong Province) were obtained from the National Geographic Information Resource Sharing Service Platform.^1^ Zibo City comprises nine districts. To analyze spatial distribution patterns, the city was divided into three geographic regions: Southern (Yiyuan County, Boshan District, Zichuan District), Central (Zhoucun District, Zhangdian District, High-Tech Development Zone, Linzi District), and Northern (Gaoging County, Huantai County), as illustrated in Figure 1. The “district/county” level was selected as the study unit.

### Global spatial autocorrelation analysis

Spatial autocorrelation analysis included global and local methods. Moran’s I, proposed by Moran in 1948, measures the similarity of attribute values across adjacent spatial units (25–27). Using Equation 1, Moran’s I was calculated based on the covariance of statistical correlation coefficients:


(1) $$I=\frac{n}{\sum_{i=1}^{n}\sum_{j=1}^{n}\mathrm{Wij}}\times \frac{\sum_{i=1}^{n}\sum_{j=1}^{n}\mathrm{Wij}(\mathrm{Xi}-\bar{X})(\mathrm{Xj}-\bar{X})}{\sum_{i=1}^{n}\;{\left(\mathrm{Xi}-\bar{X}\right)}^{2}}$$

where $W_{\mathrm{ij}}$ is the spatial weight matrix reflecting adjacency, $X_{i}$ and $X_{j}$ are attribute values, and $\bar{X}$ is the mean. Moran’s I > 0 indicates positive spatial correlation, I < 0 negative correlation, and I = 0 random spatial distribution. The standardized statistic $Z(I)=[I-E(I)]/\sqrt{\mathrm{Var}(I)}$ was used to assess significance. A spatial autocorrelation was deemed statistically significant if |Z| > 1.96 and *p* < 0.05. This study applied global spatial autocorrelation to characterize the overall spatial distribution of brucellosis in Zibo (2006–2024).

### Temporal-spatial scan statistics

Retrospective spatiotemporal scan statistics (Kulldorff’s method) were used to detect non-random spatiotemporal clusters of brucellosis from January 2006 to December 2024 (28–30). A cylindrical scanning window (circular geographic base, height representing time) moved across the study area and period, generating infinite potential clusters. The null hypothesis assumed equal relative risk (RR) inside and outside the window, while the alternative posited higher RR within the window. Under a Poisson distribution assumption, the log-likelihood ratio (LLR) for each window was calculated as Equation (2):


(2) $$\mathrm{LLR}=\log{\left(c/n\right)}^{c}{\left[(C-c)/(C-c)\right]}^{\left(C-c\right)}$$

where C is the total case count, and c and *n* represent observed and expected cases within the window, respectively. Monte Carlo simulations (*α* = 0.05, 999 repetitions) estimated *p*-values. The window with the highest LLR was defined as the most likely cluster, while others with statistical significance were secondary clusters. Spatial and temporal window sizes were capped at 25% of the population-at-risk and 30 days, respectively.

A brucellosis database (2006–2024) was constructed using Microsoft Excel 2016. Descriptive statistics summarized epidemiological characteristics. Seasonal decomposition analyzed incidence trends. ArcGIS 10.8 performed global spatial autocorrelation and mapped spatial distributions. SaTScan 9.7 identified spatiotemporal clusters.

### ARIMA model

The ARIMA model, which stands for Autoregressive Integrated Moving Average model, is suited to data exhibiting distinct trends, seasonality or autocorrelation characteristics. Capable of effectively handling non-stationary time series, the ARIMA model integrates three components—autoregressive (AR), differencing (I) and moving average (MA) elements—to capture lagged relationships within the sequence and random fluctuations. The ARIMA model formula is expressed as:


$$\Phi (B)\nabla {\mathrm{dx}}_{t}=\Theta (B)\varepsilon_{t}$$

$$E(\varepsilon_{t})=0,\mathrm{Var}(\varepsilon_{t})=\sigma_{\varepsilon }^{2},E(\varepsilon_{t}\varepsilon_{s})=0,s\neq t$$

$$E(x_{s}\varepsilon_{t})=0,\forall_{s}<t$$

The above expression can be concisely denoted as $\nabla^{d}x_{t}=\frac{\Theta (B)}{\Phi (B)}$ , where { $\varepsilon_{t}$ } represents a zero-mean white noise sequence. The ARIMA model is defined by three parameters: p, d and q, corresponding to the order of the autoregressive component, the degree of differencing, and the order of the moving average component, respectively. If the time series exhibits seasonal patterns, a multiplicative seasonal model [ARIMA (p, d, q) (P, D, Q) s]. Here, P denotes the seasonal autoregressive order, D represents the seasonal differencing order, Q indicates the seasonal moving average order, and s specifies the seasonal period.

## Results

### Demographic distribution

In 2006, Zibo City began reporting human brucellosis cases through the Infectious Disease Reporting Information Management System of the Chinese Center for Disease Control and Prevention. From 2006 to 2024, a total of 2,176 human brucellosis cases were reported in Zibo City, with an average annual incidence rate of 2.50 per 100,000 population. Among the 2,176 cases, 1,525 were male and 651 were female, resulting in a male-to-female ratio of 2.34:1. Middle-aged and older adult individuals (35–74 years old) were the primary affected group, accounting for 86.76% (1,888/2,176) of all reported cases. In terms of occupational distribution, farmers were the most affected group, representing 83.04% (1,807/2,176) of cases, followed by individuals engaged in household work or unemployed (4.69%, 102/2,176).

### Temporal distribution

**Figure 2:**
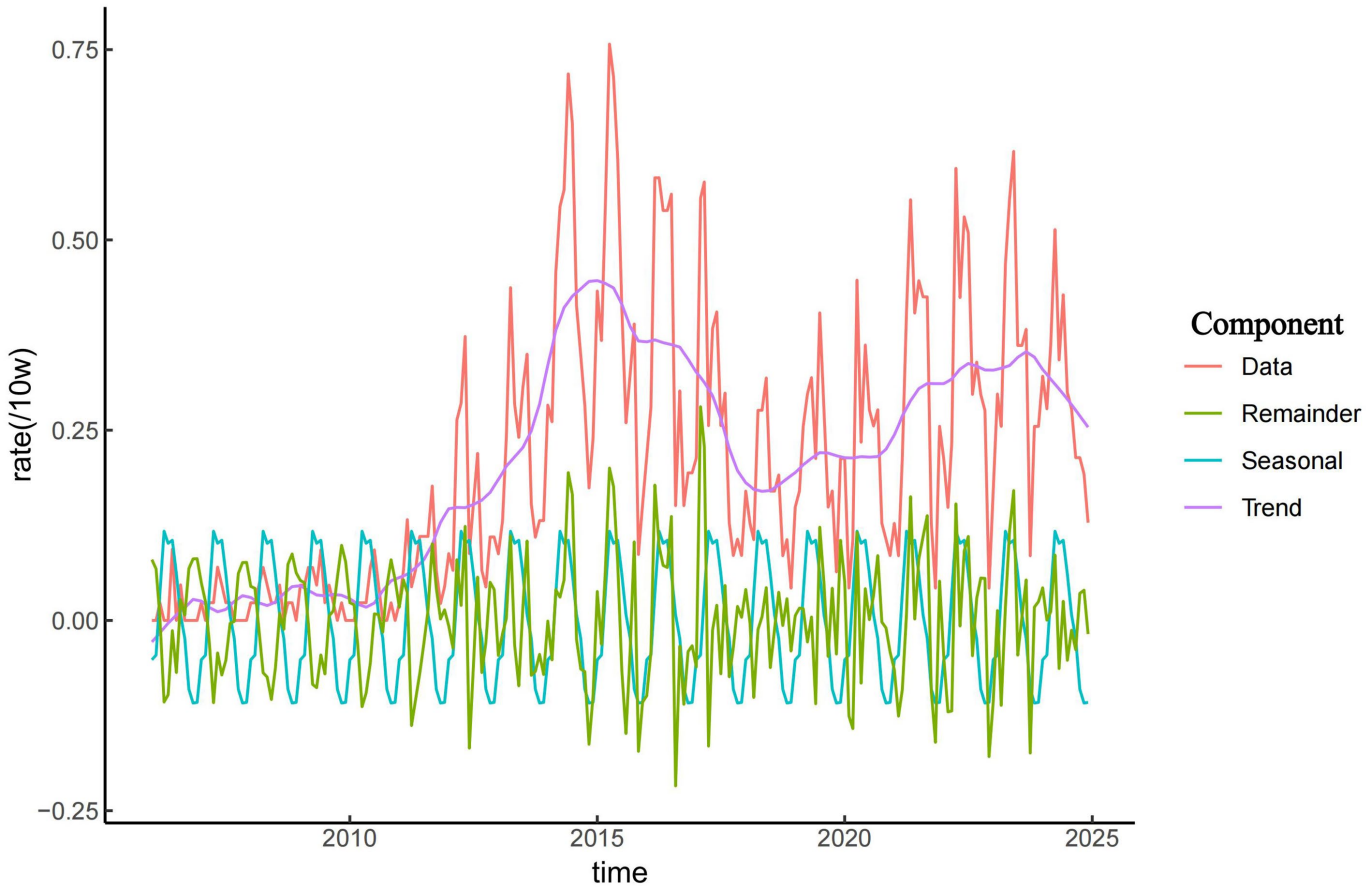
Seasonal and long-term trends of brucellosis in Zibo City, 2006–2024.

As shown in Figure 2, the incidence of brucellosis in Zibo City exhibited a long-term upward trend from 2006 to 2024. From 2006 to 2011, the incidence remained relatively low, with fewer than 20 cases reported annually and an incidence rate ranging from 0.16 to 0.30 per 100,000 population. Since 2011, the overall incidence of brucellosis in Zibo City showed an initial increase followed by a decline, peaking in 2015 at 5.24 per 100,000 population. During the period from 2011 to 2015, the average annual incidence rate was 3.13 per 100,000, reflecting a year-by-year upward trend. The results in Figure 2 also indicate a distinct seasonal pattern in brucellosis cases in Zibo City. The majority of reported cases occurred between March and September, accounting for 74.59% (1,623/2,176) of total cases, with the annual peak observed between April and June. In contrast, November had the lowest number of cases, and fewer cases were reported from October to February.

### Regional distribution

**Figure 3:**
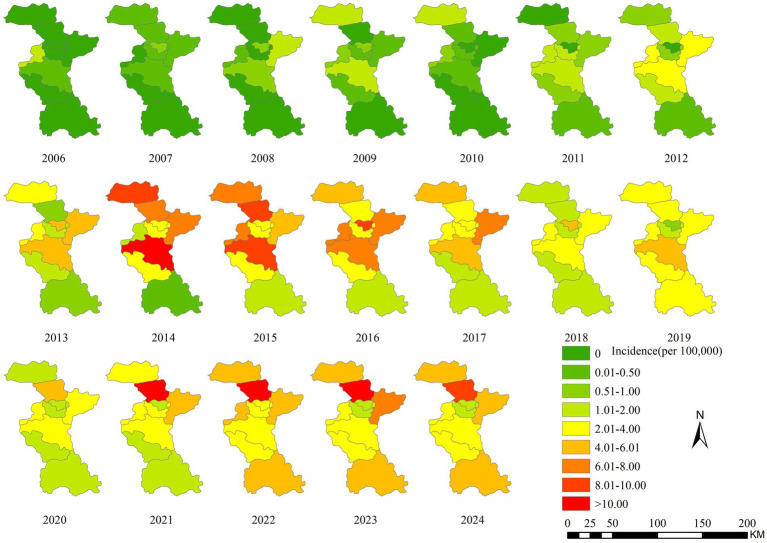
Temporal-spatial distribution map of brucellosis incidence in Zibo City from 2006 to 2021.

From 2006 to 2024, the incidence of human brucellosis in Zibo City gradually expanded from the northern and central regions to the southern areas. Cases of brucellosis first appeared in Zichuan District, located in the central part of Zibo, followed by multiple neighboring districts and counties in the central region, including Zhangdian District, Linzi District, and the High-Tech Development Zone. Since 2011, the number of human brucellosis cases in Zibo City increased rapidly, with the epidemic spreading from the northern and central regions to the southern districts of Boshan and Zichuan. By 2013, brucellosis had become endemic across all districts of Zibo City, with the number of reporting districts increasing from 2 in 2006 to 9 in 2013. The primary high-risk areas for brucellosis in Zibo City included Huantai County, Gaoging County, Linzi District, Zichuan District, Yiyuan County, and Boshan District, while fewer cases were reported in Zhangdian District and the High-Tech Development Zone. Huantai County and Zichuan District consistently reported higher rates of brucellosis, with incidence rates significantly exceeding those of other regions in most years. In 2014 and 2015, Zichuan District recorded the highest incidence of brucellosis in Zibo City. However, since 2021, Huantai County has surpassed all other districts, reporting the highest incidence rates in the city. Figure 3 illustrates the spatiotemporal distribution of brucellosis incidence in Zibo City from 2006 to 2024.

### Global spatial autocorrelation

The global spatial autocorrelation analysis of annual brucellosis incidence in Zibo City from 2006 to 2024 (conducted at the district/county level) revealed positive Moran’s I values for 2009 and 2012–2024 (|Z| > 1.96, *p* < 0.05), indicating non-random distribution and the presence of positive spatial correlation. Furthermore, the incidence of brucellosis generally exhibited characteristics of spatial clustering. The degree of clustering peaked in 2014 (Moran’s I = 0.38) and reached its lowest point in 2021 (Moran’s I = 0.11). No spatial autocorrelation was observed from 2006 to 2008 and 2010 to 2011 (|Z| < 1.96, *p* > 0.05), suggesting the absence of clustering trends during these years. The Moran’s I, Z-values, and *p*-values for 2006–2024 are summarized in Table 1.

**Table 1:** Global autocorrelation of Moran’s I values of brucellosis in Zibo City from 2006 to 2021.

Year	Moran’s I	*Z* sore	*p* value	Aggregation
2006	−0.06	0.41	0.68	No
2007	0.02	0.81	0.42	No
2008	−0.21	−0.46	0.65	No
2009	0.24	4.62	<0.05	Yes
2010	0.08	1.15	0.25	No
2011	−0.11	0.08	0.93	No
2012	0.18	2.87	<0.05	Yes
2013	0.32	5.01	<0.05	Yes
2014	0.38	4.92	<0.05	Yes
2015	0.14	3.08	<0.05	Yes
2016	0.31	3.94	<0.05	Yes
2017	0.15	2.15	<0.05	Yes
2018	0.31	3.85	<0.05	Yes
2019	0.26	2.15	<0.05	Yes
2020	0.28	4.85	<0.05	Yes
2021	0.11	2.07	<0.05	Yes
2022	0.32	5.16	<0.05	Yes
2023	0.19	3.71	<0.05	Yes
2024	0.26	4.69	<0.05	Yes

### Temporal-spatial aggregation

**Figure 4:**
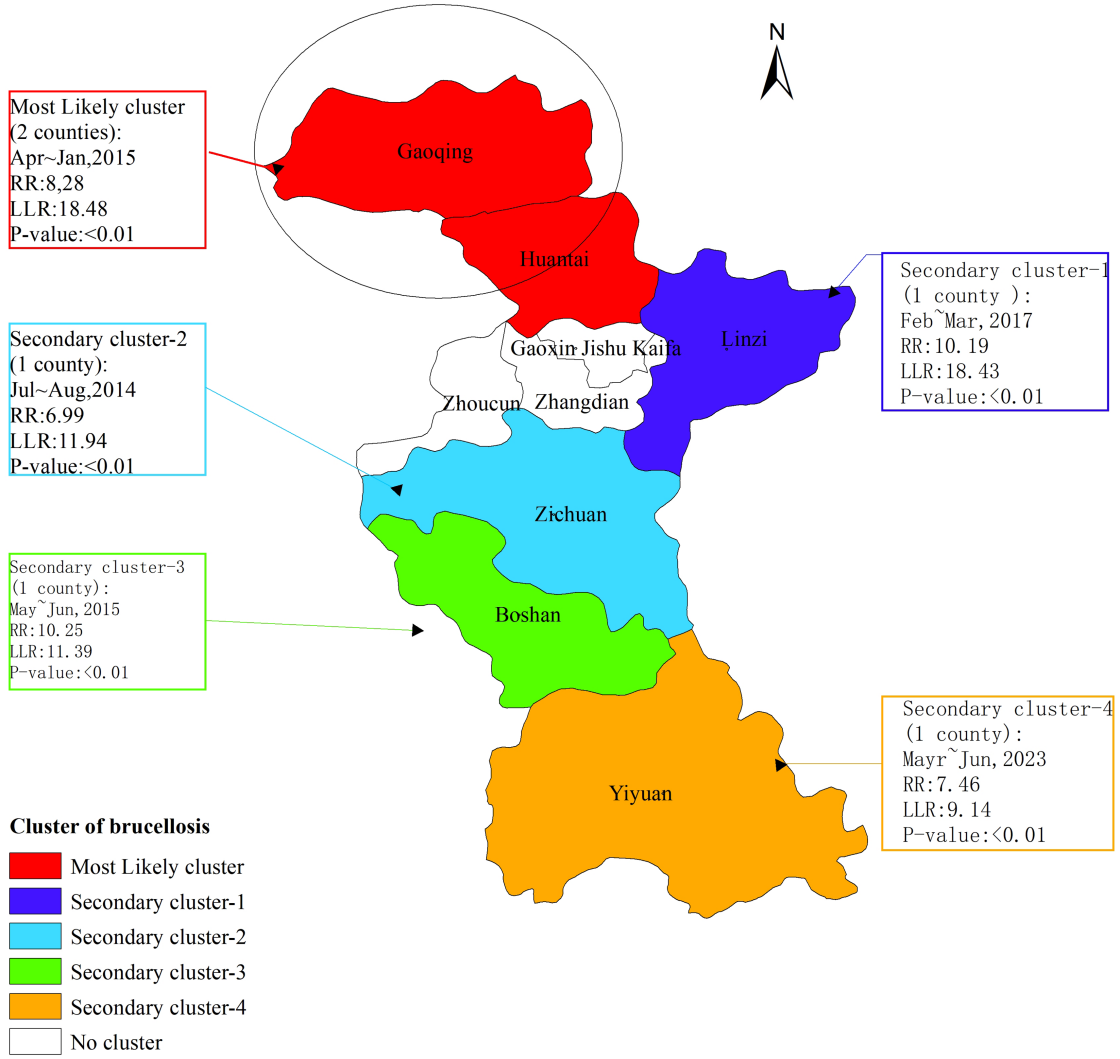
Temporal-spatial aggregation of brucellosis in Zibo City from 2006 to 2024.

The temporal-spatial clustering analysis revealed that the clusters of brucellosis outbreaks in Zibo City from 2006 to 2024 were primarily concentrated in the northern part of the city. The temporal distribution of these clusters coincided with the peak periods of brucellosis outbreaks. The analysis identified one most likely cluster and four secondary clusters. The most likely cluster was located in the northern region of Zibo, encompassing Gaoging County and Huantai County, with a radius of 26.25 kilometers. The high-risk period for this cluster was from July to August 2014 (log-likelihood ratio [LLR] = 11.94, relative risk [RR] = 6.99, *p* < 0.01). The four secondary clusters were distributed across the eastern and southern districts of Zibo: One in Linzi District, with a high-risk period from April to May 2017; one in Zichuan District, with a high-risk period from July to August 2014; one in Boshan District, with a high-risk period from May to June 2015; and one in Yiyuan County, with a high-risk period from May to June 2023 (Figure 4).

### The ARIMA model forecast results

**Figure 5:**
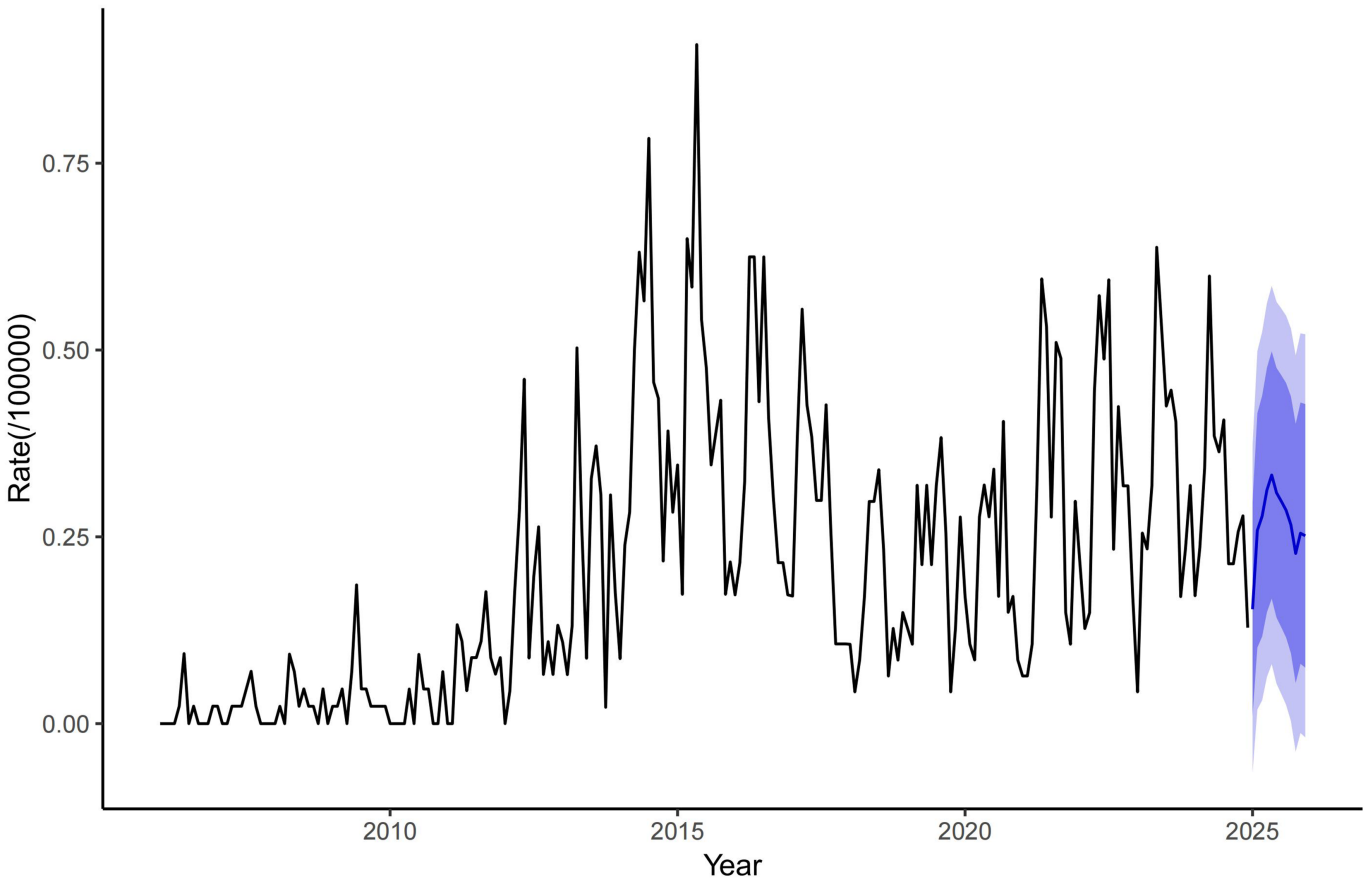
Forecast of brucellosis incidence rate in Zibo City.

A database was established using the monthly incidence rate of brucellosis in Zibo City from January 2006 to December 2024. Model parameter auto-identification, performed using R software version 4.4.3, identified the optimal model as the ARIMA (1,1,1) (0,0,2) model, with an AIC of −342.34 and BIC of −325.21. The Ljung-Box test yielded a $\chi^{2}$ statistic of 41.484 (*p* = 0.989), indicating no statistically significant differences in the Q-statistic and confirming that the residual series is white noise. The stationary *R*^2^value of 0.311 demonstrates the model is ability to explain variance in the original data, validating its suitability for predicting disease incidence trends. Based on the ARIMA (1,1,1) (0,0,2) model, the monthly brucellosis incidence rate in Zibo City for 2025 was fitted and forecasted (Figure 5).

## Discussion

Brucellosis, as a major global public health threat, presents particularly severe challenges for prevention and control in developing countries. This study focuses on the epidemiological distribution characteristics of the disease, systematically analyzing population susceptibility, spatiotemporal transmission patterns, and geographic clustering to establish a theoretical foundation for a scientific prevention and control framework. The research findings provide critical guidance for optimizing region-specific control strategies and advancing precision management of infectious diseases. They offer evidence-based insights for designing tiered intervention measures, especially in regions with limited healthcare resources (31, 32).

Our findings reveal significant age-related differences in brucellosis incidence within the study population. The majority of reported cases in Zibo City involved middle-aged and older adult individuals (35–74 years old), predominantly farmers. This age distribution is closely tied to the critical role of this demographic in household labor. In rural Chinese families, most middle-aged and older adult individuals (35–74 years old) are engaged in livestock rearing or related activities, such as livestock trading, slaughtering, and the processing of animal byproducts, including hides, dairy, and meat—particularly in regions with active livestock trading and slaughtering industries. This occupational exposure contributes to the higher incidence of brucellosis in this age group, highlighting the elevated risk of infection among middle-aged and older adult occupational populations. Cases in other age groups may be linked to the consumption of unpasteurized dairy products (33–37).

The epidemic trend of brucellosis in Zibo City aligns with the national pattern. From 2006 to 2024, the incidence of brucellosis in Zibo City increased rapidly. The number of districts reporting brucellosis cases expanded from 2 in 2006 to covering all districts by 2024, with Huantai County, Gaoging County, and Linzi District reporting significantly higher incidence rates compared to other areas. This is primarily attributed to the frequent cattle and sheep farming and trading activities in these regions, which heighten occupational exposure risks and contribute to the escalating severity of brucellosis outbreaks in Zibo City (38, 39).

The fluctuations in brucellosis cases in Zibo City from 2006 to 2024 resulted from the dynamic interplay of multiple factors. The lower case numbers between 2006 and 2010 may be attributed to reduced human-animal contact due to the transition toward intensive farming practices, strict disease prevention policies (e.g., compulsory vaccination and quarantine), and potential underreporting caused by an underdeveloped surveillance system. The surge in cases from 2011 to 2015 was linked to rapid livestock industry expansion involving unquarantined animals, relaxed biosecurity measures, frequent cross-regional live animal trade, and improved diagnostic and reporting capabilities. Post-2016, the decline in cases stemmed from strengthened government interventions (e.g., culling infected livestock, standardized farming practices), heightened public awareness of protective measures, reduced direct human-animal exposure through scaled-up industrial farming, and natural epidemiological cycles coupled with diminished susceptible populations. To consolidate control achievements, continuous efforts are needed to enhance source control in animal populations, safeguard high-risk occupational groups, and implement intelligent monitoring systems to prevent resurgence.

Seasonal decomposition analysis revealed that from 2006 to 2024, the incidence of brucellosis in Zibo City showed a year-by-year upward trend, with cases predominantly concentrated between March and September and fewer cases reported in other months. Additionally, brucellosis exhibited a distinct seasonal pattern, peaking from April to June. This seasonal surge is attributed to the lambing and calving season of sheep and cattle during spring and summer, during which occupational groups involved in lambing, calving, and livestock care are at higher risk of infection. Furthermore, livestock trading and slaughtering activities are most active from January to February. Occupational exposure to infected animals during this period typically leads to an incubation period (ranging from 3 weeks to 6 months, averaging 1 month), with symptoms manifesting around March (40, 41).

The distribution of cases gradually expanded from the northern and central districts of Zibo City to the southern regions. This spread is driven by the well-developed livestock industry in Gaoging County and Huantai County in northern Zibo, where numerous farms and small-scale breeders operate. Additionally, Linzi District, home to a Hui ethnic community engaged in livestock trading, experiences frequent movement of people and animals between neighboring cities, particularly Binzhou City. These factors have contributed to the worsening brucellosis epidemic in northern and central Zibo, with subsequent diffusion to southern districts. These findings underscore the need to strengthen brucellosis prevention and control measures in northern and southern Zibo, including standardizing livestock farming and trading practices and enhancing quarantine measures for imported animals. Furthermore, molecular epidemiological studies could help identify the sources of infection in central Zibo, enabling targeted intervention strategies (42).

Global autocorrelation analysis reveals that from 2009 and 2012 to 2024, Brucellosis in Zibo City exhibited significant spatial clustering at the county level. Spatial–temporal cluster analysis identified one primary cluster and four secondary clusters, which essentially cover the entire area of Zibo, indicating that the spread of Brucellosis is a result of combined spatial–temporal domains rather than mere spatial or temporal variations. Additionally, the relative risk (RR) values for these four clusters range from 6.99 to 10.25 (*p* < 0.05), suggesting that the risk of human Brucellosis in these cluster areas is significantly higher than in other parts of Zibo City, reflecting the high incidence characteristics of Brucellosis in Zibo. The primary cluster is located in Gaoqing County and Huantai County of Zibo City, with the clustering time concentrated between April and May 2015. This cluster is attributed to the increased risk of exposure to infected sheep among occupational groups in these areas through activities such as breeding, trading, and slaughtering, as well as the rising incidence of Brucellosis in recent years, indicating that prevention and control measures in these regions still need to be strengthened (43).

Our findings indicate a connection between high-risk areas and clustering characteristics, particularly in non-endemic regions with high incidence rates. Therefore, the dynamic changes in high-risk areas of Brucellosis in Zibo City may be closely related to disease outbreaks. In recent years, the number of Brucellosis cases in Zibo has increased rapidly, with multiple outbreaks occurring in livestock slaughterhouses, breeding farms, and trading centers. We recommend that governments at all levels establish joint prevention and control mechanisms, implementing strict Brucellosis control measures in both animals and humans to meet the requirements of disease prevention and control.

According to ARIMA model predictions, the monthly incidence rate of brucellosis in Zibo City has declined steadily from 0.75/100,000 in 2010 to near-zero levels by 2020 and is projected to remain extremely low through 2025, indicating the significant effectiveness of current prevention measures such as animal quarantine, epidemic surveillance, and public education. However, the model does not account for potential risks like sudden outbreaks, declining population immunity, or dynamic changes in animal husbandry, necessitating vigilance against imported cases or localized epidemics. Recommendations include strengthening multi-sectoral collaborative monitoring, prioritizing protection for livestock workers, promoting public health education, improving emergency response mechanisms, and integrating machine learning models with serological studies to refine predictive accuracy and prevention strategies. These efforts aim to prevent complacency due to prolonged low incidence and consolidate the achievements in disease control.

This study has some limitations. First, undiagnosed, unreported, or cases that did not meet the case definition criteria were not included in the analysis, which may lead to an underestimation of the incidence of Brucellosis in Zibo City. Second, we used adjacency criteria for spatial autocorrelation analysis, with the scale of adjacent areas adjusted through a spatial weight matrix. This may cause variations in correlation coefficients when calculating Moran’s I, affecting the final clustering results and introducing selection bias. Third, the spatial–temporal scan statistical method used to detect clusters in different spatial and temporal periods relies solely on circular spatial scanning and cylindrical spatial–temporal scanning, without considering irregular spatial patterns.

## Conclusion

This study contributes to identifying high-risk populations, areas, and time periods for Brucellosis, providing valuable insights for decision-making by relevant authorities. Based on the distribution characteristics of Brucellosis outbreaks, we recommend strengthening detection and continuous monitoring efforts in the northern counties of Zibo and implementing effective Brucellosis prevention and control strategies in the central regions of Zibo, where the incidence rate is relatively low. At the same time, it is essential to enhance the epidemic response capacity in high-risk areas to prevent further spread of Brucellosis.

## Data Availability

The raw data supporting the conclusions of this article will be made available by the authors, without undue reservation.

## Footnotes

^1^ https://www.web-map.cn
